# The Molecular Gut-Brain Axis in Early Brain Development

**DOI:** 10.3390/ijms232315389

**Published:** 2022-12-06

**Authors:** Fahim Muhammad, Bufang Fan, Ruoxi Wang, Jiayan Ren, Shuhui Jia, Liping Wang, Zuxin Chen, Xin-An Liu

**Affiliations:** 1 Shenzhen Key Laboratory of Drug Addiction, Shenzhen Neher Neural Plasticity Laboratory, The Brain Cognition and Brain Disease Institute, Shenzhen Institute of Advanced Technology, Chinese Academy of Sciences, Shenzhen 518055, China; 2 Shenzhen-Hong Kong Institute of Brain Science-Shenzhen Fundamental Research Institutions, Shenzhen 518055, China; 3 Guangdong Provincial Key Laboratory of Brain Connectome and Behavior, CAS Key Laboratory of Brain Connectome and Manipulation, Brain Cognition and Brain Disease Institute (BCBDI), Shenzhen Institute of Advanced Technology, Chinese Academy of Sciences, Shenzhen 518055, China; 4 University of Chinese Academy of Sciences, Beijing 100049, China

**Keywords:** gut-brain axis, molecules, neurodevelopment, vagus nerve, epigenetics

## Abstract

Millions of nerves, immune factors, and hormones in the circulatory system connect the gut and the brain. In bidirectional communication, the gut microbiota play a crucial role in the gut-brain axis (GBA), wherein microbial metabolites of the gut microbiota regulate intestinal homeostasis, thereby influencing brain activity. Dynamic changes are observed in gut microbiota as well as during brain development. Altering the gut microbiota could serve as a therapeutic target for treating abnormalities associated with brain development. Neurophysiological development and immune regulatory disorders are affected by changes that occur in gut microbiota composition and function. The molecular aspects relevant to the GBA could help develop targeted therapies for neurodevelopmental diseases. Herein, we review the findings of recent studies on the role of the GBA in its underlying molecular mechanisms in the early stages of brain development. Furthermore, we discuss the bidirectional regulation of gut microbiota from mother to infant and the potential signaling pathways and roles of posttranscriptional modifications in brain functions. Our review summarizes the role of molecular GBA in early brain development and related disorders, providing cues for novel therapeutic targets.

## 1. Introduction

The human gut harbours trillions of commensal microbes crucial for maintaining host immunity and cellular homeostasis [1]. “Gut microbiome” refers to the gut microbial genome. This microbial genome encodes structural proteins and enzymes responsible for cellular function in the gut. Microbes and their metabolic products help maintain nutrient balance, regulate cellular metabolism and the immune system, build mucosal barriers, and destroy pathogens [2]. Gut microbes regulate endocrine function by restoring insulin sensitivity and decreasing intestinal inflammation [3]. Age-related disorders, such as obesity [4], arthritis [5], type 2 diabetes [6], hypertension [7], metabolic disorders [8], and stroke [9], are associated with the gut microbiota. Moreover, the gut microbiota regulate the two-way communication between the gut and the brain. Visceral organs constantly communicate with the brain to maintain the normal physiological functioning of the body and internal homeostasis. The gut-brain axis (GBA) plays a crucial role in the interoceptive response circle. The GBA is responsible for the bidirectional communication between the central nervous system (CNS) and the enteric nervous system (ENS). The GBA is a mediator that connects the emotional and cognitive areas of the brain to the gut [10]. Previous studies have reported that the GBA regulates mood and cognitive function associated with the CNS and ENS. Therefore, recent studies have investigated the clinical applications of GBA for treating neurodevelopmental disorders, such as autism spectrum disorder (ASD) [11]. This GBA association involves immune, endocrine, and neural regulation. A previous study reported that the GBA comprises several essential pathways, such as the neuroendocrine system, autonomic nervous system, and immune regulation system, which are interlinked by the vagus nerve (VN) [12]. The VN, a primary nerve in the parasympathetic nervous system (PNS), regulates involuntary mechanisms such as immunity, digestion, respiratory rate, and the cardiac cycle. Brain dysfunction occurs due to gut microbiota disruption, resulting in abnormal behaviours. It has been reported that gut microbiota can regulate brain activity by activating the hypothalamic-pituitary-adrenal (HPA) axis through the synthesis and release of various neurotransmitters, neuroactive immune cells, and neuropeptides [13], thereby influencing anxiety- and depression-like behaviours. These studies establish the crucial role of gut microbiota in brain development. Our review provides an overview of the existing evidence on the role of gut microbiota and their metabolites in preventing and treating several neurological diseases. Furthermore, we have featured the role that gut microbiota-related therapeutics could play in ameliorating these disorders.

## 2. Gut Microbiota and Metabolic Factors

The role of gut microbiota in several disorders, including obesity, neurodegeneration, and cancer, has recently gained attention. The gut harbours various microorganisms that can elicit considerable responses. Similarly, after the fetus’s birth, the initial microbiota form and grow alongside the host [14]. Although the gut microbiota are easily agitated during the initial years of postnatal development, consistent microbiota are observed over time. Genetic and environmental factors, including food, antibiotics, and exposure to other microbes, control the fetus’s early gut microbiota composition [15]. Thus, a healthy adult gut microbiota is highly adapted to the host and the host’s environment. It transmits biochemical and metabolic functionality critical to brain development (Table 1). The adult gut microbiota are relatively stable but retain some tractability in response to internal and external environmental factors. Food, antibiotics, infections, and anxiety affect the gut microbiota and other human body parts [16]. The association between genetic manipulation, immune system regulation, and aging requires additional research to obtain a more detailed understanding [17].

## 3. The Roles of the VN in the GBA and Brain Development

The VN mediates the sixth sense and is an essential component of the PNS due to its role in interceptive awareness. VN is the parasympathetic link between the gut and the brain [37]. The VN serves as the neural axis establishing the GBA phenomena [37]. It can identify microorganisms in the gut and transmit this information to the CNS, where the information is integrated, following which it generates an adapted response. The VN has the potential to alleviate eating disorders such as compulsive eating [38]. The VN is a mixed nerve and transmits anti-inflammatory signals that are exhibited through its fibers at the GBA interface. Targeting the VN could restore homeostasis in neuronal cells, thereby controlling brain development and delaying age-related degenerative disorders [39]. VN stimulation has been used in clinical settings to treat depression and epilepsy. Furthermore, the anti-inflammatory and antiepileptic effects via stimulating the VN should be of interest in the clinical applications. The VN helps control serotonin levels in the stomach and regulates the response of serotonin to brain development under the influence of the GBA [37]. The tail suspension test confirmed that selective serotonin reuptake inhibitors (SSRIs) could not treat depression in C57BL mice. This finding suggests that SSRIs are ineffective without the support of the VN. VN functional abnormalities result in neurodevelopmental disorders [40]. Therefore, the VN plays a crucial role in gut-brain interactions.

## 4. The Molecular Mechanisms of Gut Microbiota on Brain Development

A recent study on how mothers and fetuses interact evaluated various aspects such as nutrition, gut metabolites, and factors that help improve the mother’s immune system [41]. Maternal gut metabolites cross the placenta and circulate in the fetal blood circulation. The mother is hyperphagic and has fat deposits during the early stages of pregnancy. Minimal fetal growth occurs in this instance. Optimal fetal growth occurs during the third trimester of pregnancy when the maternal metabolism is at its peak [42]. During pregnancy, the maternal gut microbiota serves as an essential catabolic regulator and immune enhancer, which can break down carbohydrates to produce fatty acids, amino acids, and vitamins. Amino acids and vitamins play a crucial role in metabolic activity, immunity, and fetal development [43]. Metabolic dysregulations in the maternal gut microbiota impede physiological and mental development in the fetus [44]. Nonetheless, several studies have reported that maternal gut microbiota play a crucial role in regulating the fetal immune system and postnatal brain development. Another reason for metabolic dysregulations in maternal gut microbiota could be variations in the composition of the gut microbiota [45], vaginal microbiota [46], and oral microbiota [47], along with gestational diabetes. Pregnancy is a critical window for regulating the gut microbiota, and the factors influencing the maternal microbiota involved in the intergenerational communication between mother and offspring remain complex. Significant changes occur in various microbial species during pregnancy, and specific microbe functions and their interactions with the host brain must be carefully considered. It is important to understand the factors that cause changes in the maternal microbiota to develop effective plans for monitoring maternal and foetal health during pregnancy based on GBA intergenerational transmission and its role in brain development [48].

### 4.1. The Dynamic Gut Microbiota and Brain Development

**Figure 1:**
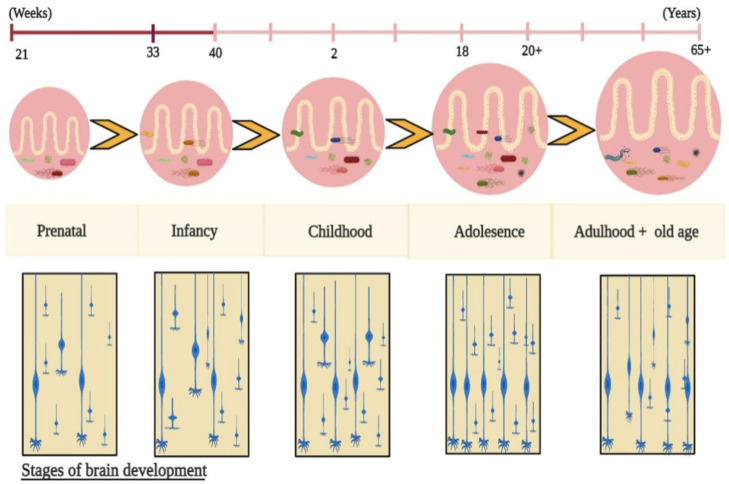
Evolution of the gut microbiota along with synaptogenesis throughout the lifespan. After birth, infant gut microbiota are established, and they are exposed to multiple environmental factors which influence the intestinal microbiota composition, thereby controlling physiological growth, brain development, and behaviours via gut metabolites.

As previously stated, the oral, gut, and vaginal microbiota change over time [49], and these changes are associated with several factors, such as food, antibiotics, stress, and host genetics [50]. Romero et al. reported that the gut microbiota of healthy pregnant women are more vulnerable than the gut microbiota of healthy nonpregnant women. Understanding how the maternal intestinal microbiota impact fetal growth is fundamental to knowing how maternal gut microbiota affect foetal physical and mental growth [51]. This allows researchers to assess the maternal gut microbiota for evaluating fetal physiological growth and brain development [52]. During pregnancy, the maternal gut microbiota disrupts fetal brain development far more than vaginal, placental, uterine, and gastrointestinal microbiota. Molecular approaches, such as 16S ribosomal ribonucleic acid (RNA) gene sequencing and metagenomics, have significantly increased our understanding of the microbiome at the maternal-fetal interface during intrauterine life [53]. Abrupt exposure to environmental and genetic factors after birth plays a role in establishing healthy growth of foetal microbiota [54]. During the initial years of life, infant gut microbiota undergo significant changes in their taxonomic composition [55]. With age, the infant is exposed to new environments and is colonized by new microbes, increasing the infant’s gut microbial diversity. In the initial stages, the infant microbiota is more diverse than the maternal microbiota. Exposure to various environmental microbes and factors, such as diet, location, and lifestyle, gradually decreases the variations found in infant microbiota. With age, the gut microbiota are constantly changing along with synaptogenesis throughout the lifespan (Figure 1), but here we only focus on the early developmental stages. The infant acquires a new microbial family due to changes in the gut microbiota and brain developmental stages. Recent study found that a lower gut microbial diversity was associated with a higher risk of neurodevelopmental delay for the small for gestational age (SGA) infants compared with the appropriate for gestational age (AGA) infants [56].

### 4.2. The Dynamic Gut Microbiota and Brain Structure Developments

Braniste et al. reported that germ-free mice displayed increased BBB permeability that initiated with the fetus’s intrauterine life and was maintained after birth and during adulthood. This BBB permeability was associated with reduced expression of the tight junction proteins occludin and claudin-5, which are known to regulate barrier function in endothelial tissues [19]. The results suggested that the maternal microbiota influences BBB development in the uterus. Moreover, evidence from germ-free mice showed that the maternal microbiome can influence the maturation of embryonic microglia, with microglia being more profoundly perturbed in male embryos and female adults [57]. Depletion of the maternal microbiome decreased the expression of genes involved in axonogenesis, resulting in deficient thalamocortical axons and impaired outgrowth of thalamic axons in response to cell-extrinsic factors in the offspring. Select microbiota-dependent metabolites or maternal supplementation promoted axon outgrowth and rescued the deficiencies in fetal thalamocortical axons [58]. These results suggest that the maternal microbiota is important for the axonogenesis and neurodevelopment of the fetal brain.

### 4.3. The Gut Microbiota-Derived Neurotransmitters in Brain Development

Several neurotransmitters derived from gut microbiota are known to modulate peripheral and central sensitization and, in turn, mediate neurodevelopment (Table 2). In the CNS, gut microbiota-derived mediators may trigger neuroinflammation, which involves infiltrating immune cells and activating cells in the BBB. **Catecholamines**, such as dopamine and norepinephrine, regulate several central and peripheral nervous system functions, including cognitive ability, emotion, and intestinal movement [59]. In the gut, dopamine and norepinephrine are mainly present in the colon cavity. It has been found that the catecholamine level of germ-free mice is lower than that of mice without specific pathogens; a previous study showed that certain bacteria in Staphylococcus can produce dopamine through staphylococcus aromatic amino acid decarboxylase [60]. In addition, dopamine is also found in the biomasses of *Staphylococcus aureus*, *Bacillus cereus*, *Proteus vulgaris, Serratia marcescens,* and *Escherichia coli* [61]. **Dopamine** regulates the function of immune cells and activates cytokines produced by T cells [62]. In the CNS, dopamine regulates nitric oxide synthesis and microglial cell migration [63,64]. Decreased norepinephrine levels are associated with depression, anxiety, and post-traumatic stress disorder [65]. **Noradrenaline** is found in the biomasses of intestinal microbiota, including *Escherichia coli*, *Bacillus subtilis, Bacillus mycoides, Proteus vulgaris,* and *Serratia marcescens*, which indicates that these species may be able to produce noradrenaline [66]. In the brain, noradrenaline has the neuroprotective effects of inhibiting infant gene transcription and enhancing the production of brain-derived neurotrophic factor (BDNF) by microglia and astrocytes, which can further promote neuronal survival [67,68]. In addition, norepinephrine can regulate excitability and neuronal responses [68]. **Lactic acid bacteria** (LAB) can produce γ-aminobutyric acid (GABA) from GABA-rich fermented foods and beverages. LAB, such as *Lactobacillus*, *Bifidobacterium*, and *Streptococcus*, produce glutamate decarboxylase, which is used to produce GABA [69,70]. Among the 91 culturable bacteria in the human intestine, *Lactobacillus brevis* and *Bifidobacterium denticola* were found to be the most effective GABA-producing bacteria [69]. GABA is the main inhibitory neurotransmitter in the CNS, which plays an inhibitory role in the immune system through two specific receptors, GABA_A_ and GABA_B_. Several studies have shown evidence that GABA can traverse the BBB, such as through simple diffusion, solute transport via endocytosis, or carrier-mediated transport, which may allow a small amount of GABA to cross the BBB [71,72,73]. GABA plays a role in regulating the inhibition-excitation balance required for brain function, downregulating cytokines released by primary immune cells, as well as endogenous and exogenous intestinal nerve-secreted neuropeptides [74,75,76]. **GABA** also plays a role in the development of interstitial neurons of the white matter, as well as in oligodendrocyte development. However, the underlying cellular mechanisms are not yet fully understood [74]. **Serotonin** is another important neurotransmitter that transmits signals between neurons throughout the body. Results from germ-free mice showed that the level of serotonin in the blood and colon decreased [77], and the turnover rate of serotonin in the brain increased [78]. Depleted serotonin levels can be recovered by recolonizing with several bacteria, such as the association of spores-forming species. In addition, some bacterial genera, such as *Candida*, *Streptococcus*, *Escherichia coli*, *Enterococcus,* and *Pseudomonas*, can produce serotonin [79]. In mammals, serotonin derived from intestinal microbiota can play a role in the local intestine or enter the blood circulation, but it will not cross the BBB. However, it is reported that serotonin can increase the permeability of the BBB, thereby indirectly affecting brain function [80]. Serotonin regulates various immune cell functions through a variety of mechanisms and is an effective immune cell regulator in known autoimmune diseases. For example, serotonin can inhibit major histocompatibility complex class 11 (MHC class II) expression and antigen presentation in macrophages [81]. Serotonin may also reduce proinflammatory cytokines produced by macrophages and lymphocytes, such as interleukin (IL-6) and tumor necrosis factor—α (TNF- α) [82]. Serotonin produced by the gut microbiota may have a greater impact on the CNS than initially expected because the intestinal epithelium interacts with 5-HT receptor afferent fibers in vagus or dorsal root neurons [83]. Studies performed in mice have shown a dramatic increase in the development of enteric neurons after two to three weeks of treatment with serotonin 5-HT4 agonists [84]. The authors also showed that GF mice that were unable to synthesize serotonin had less neuronal development. A separate study [85] showed that neuronal dysfunction in GF mice could be reversed by recloning of the gut microbiota. 

### 4.4. The Effects of Gut Microbiota on Epigenetic Modifications and Brain Development

Epigenetic modifications affect gene expression but not base pair sequencing. The epigenetic modifications include microRNA changes, histone modifications, and deoxyribonucleic acid (DNA) methylations. DNA methylation involves the addition of a methyl group to cytosine [87]. Enzymes (methyltransferases) play a crucial role in the addition of the methyl group to DNA. Hypermethylation and hypomethylation indicate an increase and decrease in methyl group integration at the DNA level, respectively. Hypomethylation in the DNA promoter region upregulates genes [88]. It is assumed that microbiota modify DNA methylation in a genome-specific or non-genome-specific manner. For instance, the microbiota can induce oxidative stress by generating mitochondrial reactive oxygen species (ROS) via autophagy [89]. Oxidative stress modulates DNA methylation, thereby changing gene expression. The DNA base pair structural changes caused by ROS mainly comprises deletions. These structural changes inhibit DNA methylation, resulting in hypomethylation. From a bacterium-induced inflammatory response perspective, high transcription factor activity would cause altered inflammatory gene methylation [90]. Similarly, maternal gut microbiota influence epigenetic modifications during pregnancy and contribute to late fetal life. A genomic study reported that the similarity between placental and neuronal DNA methylation profiles is associated with neural development. Thus, placental DNA methylation might contribute to infant brain development and influence cognitive behaviours [91]. Experimental studies have recently reported a link between placental genes and DNA methylation patterns in fetuses using the Neonatal Intensive Care Unit Network Neurobehavioural Scale (NNNS) approach. The NNNS evaluates an infant’s neurological, social, and stress-related measures to control cognitive behaviour. The NNNS also assesses early fetal life behavioural responses and indicates brain development and cognitive functionality [92].

The Rhode Island Child Health Study technique analyzed epigenetic studies in fetuses and mothers. Using NNNS, the *hydroxysteroid 11-beta dehydrogenase 2* (*HSD11B2*) gene and several other genes are identified to evaluate the DNA methylation status [93]. *HSD11B2* genes regulate glucocorticoids in the HPA axis and the placenta. A previous study reported that decreased *HSD11B2* expression in the placenta causes fetal HPA axis dysfunction. Epigenetic analysis of 185 newborn offspring placentas revealed that the promoter region of *HSD11B2* was hypermethylated in newborns with a lower quality of movement score [94]. Similar studies reported an association between methylation configuration in the promoter region of *nuclear receptor subfamily 3 group C member 1* (*NR3C1*) with improved fetal movement, self-regulation, attention, and exhaustion. The DNA methylation interface of both genomic traits (*NR3C1* and *HSD11B2*) is associated with distinct neurobehavioural and neurodevelopmental phenotypes; however, further research is required [95].

### 4.5. The Pathways of Gut Microbiota in Brain Development

Gut microbiota dysbiosis contributes to allergy [96], asthma [97], obesity [98], inflammatory bowel diseases [99], celiac disease [99], irritable bowel syndrome [100], metabolic syndrome [101], and cardiovascular disorders [102]. Several microbial species (*Firmicutes*) have enzymes that catabolize carbohydrates from food for energy production. Thus, a higher frequency of *Firmicutes* might increase calorie absorption, resulting in gut inflammation and subsequent weight gain [103]. Evidence confirms the involvement of microbiota in nervous system disorders and other behavioural diseases [104]. The health and disease theory describes how prenatal environmental factors influence health in adulthood [105]. Overnutrition or malnutrition result in gut microbiota disbalances, causing an imbalance in microbial metabolites and neurological dysregulation, such as deficiencies in brain reward circuitry and behavioural abnormalities in fetuses. Another possibility is stress as a result of changes in the gut microbiota. Previous research has reported that stress influences changes in the microbiota structure in pregnant mice (C57BL) and in humans [106]. Certain strains of *Bifidobacterium and Lactobacillus* secrete gamma-aminobutyric acid (GABA), a key inhibitory transmitter in the CNS [107]. Changes in the GABA signaling pathway have been associated with anxiety- and depression-like disorders [108], whereas disruption of the gut microbiota affects the CNS stress response pathway. In studies of C57BL mice and the human gut microbiota model, this stress pathway has been shown to be associated with neurodevelopmental diseases [109,110]. However, observational epidemiological studies have sought to demonstrate a direct interaction link between gestational obesity and neurological disorders in the progeny [111]. Other studies in germ-free mice have reported a direct link between intestinal microbiota composition and obesity during pregnancy [112]. Reportedly, the absence of intestinal microbes protects against diet-induced obesity, and intestinal microbiota regulates prenatal brain development via interleukin (IL)-17A [113]. Mouse studies revealed that common bacteria in the ileum and caecum regulate IL-17A [114,115]. IL-17A is associated with maternal pregnancies and protects the fetus from neurodevelopmental and behavioural disorders [116]. Therefore, IL-17A could serve as an emerging therapeutic target for treating pregnancy complications.

In conclusion, gut microbiota and their metabolites are directly associated with prenatal growth. However, further research is required to confirm the role of microbiota and its metabolites as novel therapeutic targets in older women with pregnancy complications and prenatal fetal neuronal development. Nevertheless, scientific questions in this area deserve further research.

## 5. The Role of the Molecular GBA in Postnatal Brain Development and Mental Disorders

**Figure 2:**
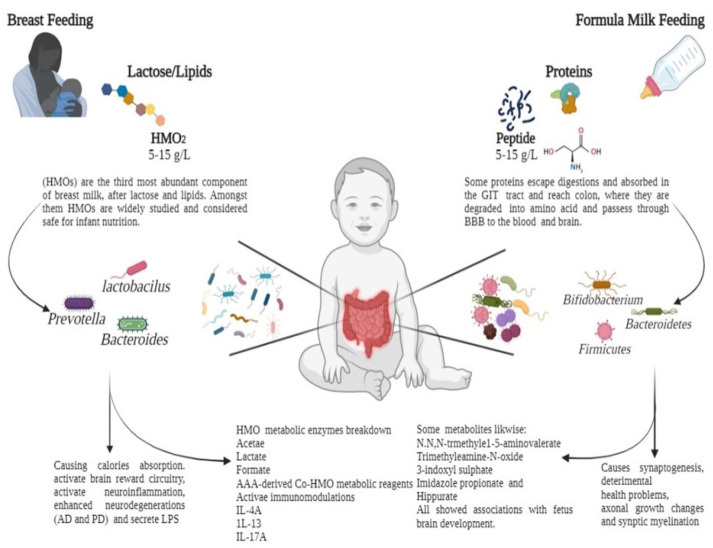
Feeding patterns influence the human gut microbiota via the fermentation of carbohydrates and proteins in early breast milk. Breast milk contains human oligosaccharides (HMOs), which pass undigested through the upper gastrointestinal (GI) tract to the colon. Here, they are digested by specific HMO-degrading microbial species (*Lactobacillus, Bifidobacterium,* and *Firmicutes*) into various useful metabolites. Upon ingestion, formula milk with excess proteins reaches the colon via the GI tract. Proteins, peptides, and individual amino acids are catabolized by gut microbiomes such as *Enterobacteriaceae* and *Clostridium* species. From breastfeeding to formula feeding, the infant is exposed to gut microbiota and metabolites, which contribute to immune regulation and neural development.

Gut microbiota regulate several biologically active molecules in extraintestinal organs, serum, and other fluids [117]. Maternal gut microbiota-induced axon outgrowth synchronises the metabolites of an infant’s brain [118]. The mechanisms by which metabolites affect an infant’s brain remain unknown [39]. Metabolites such as N, N, N-trimethyl-5-aminovalerate, trimethylamine-N-oxide, 3-indoxyl sulphate, imidazole propionate, and hippurate significantly impact infant brain development. Malnourished infants’ brains are depicted with structural changes of less white matter [119]. Therefore, early- to mid-gestation is critical because, during this period, maternal gut microbiota stimulate fetal brain development [58]. During synaptic remodelling, fetal brain changes, such as synaptogenesis, axonal growth alterations, and myelination, modify brain development [120]. Substantial synaptic and neuronal changes indicate the dynamic association and support of newly established neural circuits in early infancy [121]. Breast milk is the primary source of microbes in the infant’s gut after birth. However, the origin of the bacteria found in milk is yet to be elucidated. It is assumed that the infant’s mouth serves as the transition point from where the milk reaches the intestine via deglutition and suction, wherein the transfer of microbial colonies from the mother’s milk duct occurs. This could play a role in assembling the gut microbiota [122]. These changes in the gut microbiota and their metabolites might have long-term effects on early childhood brain activity, motor symptoms, social interactions, and cognitive behaviour [123]. It is an energy-intensive procedure that could strongly influence various environmental factors, including nutrition, social interaction, stress, and infection. After birth, infants obtain nutrients and energy from breast milk, formula milk, solid food, or metabolites in food [124]. Remarkably, several factors that impact the age-related progression of gut microbiota are majorly involved in brain development and function via the GBA [125]. However, metabolites in food might moderate these effects, influencing brain development and functions directly or indirectly (Figure 2).

The most compelling evidence for gut microbiota involvement in host psychology and physiology came from germ-free mouse experimental studies, wherein a lack of microbe-host interactions was observed since birth [126]. Lack of microbiota in these animals results in various physiological changes, including changes in gut sensory-motor functions, access to the gastric cavity via the blood-brain barrier (BBB), and enhanced immune function. Germ-free mice demonstrated significant changes in brain functionality, similar to humans [127]. Changes in neurotrophic factors, neurogenesis, serotonin levels, and amygdala neuron structure and activity explain changes in stress responses, anxiety and depression traits, and social behaviours observed in germ-free animals [128]. Several biomedical studies have demonstrated that patients with increased modifications in the gut microbiota are at a higher risk of schizophrenia, ASD, depression, and anxiety, thereby indicating an association between gut microbiota and nervous system disorders [129]. Moreover, an imbalance in the gut microbiota causes ASD in children. Several gut metabolites, including indoles, short-chain fatty acids, and lipopolysaccharides, many of which were of bacterial origin, were found in the blood samples of children with ASD. These metabolites pass through the BBB in children and cause oxidative stress, mitochondrial dysfunction, and structural changes in the amygdala, cortex, hippocampus, and cerebellum. Although the cause-effect relationship between ASD and gut microbiota is not elucidated, the diagnostic and therapeutic value of gut microbial metabolites as potential targets warrants research [130]. Similarly, it is hypothesized that gut microbiota play a role in the pathogenesis of epilepsy. The gut microbiota provide new insights into the pathogenesis and treatment of epilepsy and new therapeutic options [131]. Surprisingly, when the gut microbiota of a patient with epilepsy were transferred to germ-free animals, symptoms similar to those of epilepsy were observed in the animals, adding an epidemiological element to the GBA microbiota [132].

## 6. The Role of the GBA in the Intergenerational Effects of Brain Development

Preliminary research on pregnant women with depression and anxiety has identified a strong link between modified bacterial abundance in the gut microbiota and inflammatory responses [133]. An equal association was observed with gestational diabetes mellitus [134], preeclampsia [135], maternal obesity [136], and fetal physical and mental developmental problems [137]. Therefore, the gut microbiota and the GBA play a crucial role during the prenatal period, when the maternal and fetal microbiota are sensitive, and any changes in the microbiota could affect fetal brain development. Diet, infection, antibiotic use, and stress, particularly during the prenatal period, could cause dysbiosis and might increase the transmission of disease-causing traits from mother to offspring [58]. This mother-fetal trait-transferring relationship is known as an intergenerational relationship. Recently, an experimental study investigated the effects of the gut microbiota on ischaemic brain injuries, and several factors, such as bacterial metabolites and immune system regulation, were identified [138]. Probiotic supplements help treat disorders caused by the gut microbiota. In 20 randomized controlled trials with 2972 participants, probiotic supplementation lowered plasma glucose levels and improved insulin sensitivity and resistance, ensuring healthy foetal development [139]. Fetal growth restriction is associated with insulin resistance, increased inflammation, and glucose deficiency [140]. According to human population research and epidemiological data, intrauterine growth and maternal obesity negatively impact fetal physical and mental development [141]. However, several knowledge voids in terms of the difference in the fetal and maternal gut microbiota compositions that affect brain development exist. This study has reported growing evidence of intergenerational communications between gut microbiota and brain development. This mutual communication occurs via the GBA. Growing evidence suggests that the GBA plays a crucial role in intergenerational transmission in terms of the development and prevention of brain disorders [123,142].

## 7. Future Perspectives

It is alluring to speculate that the metabolites from the maternal gut microbiota can stimulate the offspring’s brain development during embryogenesis. Therefore, the maternal gut microbiota might play a crucial role in transgenerational brain development, improved phenotypic expressions, and endophenotypes [143]. The impact of the GBA on fetal brain developmental programming and its modulation by various microbial metabolites have been successfully demonstrated in studies. Such studies uncover the full therapeutic potential of microbial metabolites in the gut from infancy to adulthood, reducing the burden of neurological damage in future generations. Mechanistic studies that deserve further investigation include neurotransmitter release under the gut metabolites’ influence and turnover in the ventral tegmental area and substantia nigra pars compacta, synaptic plasticity impairment and recovery pathways, altered parasympathetic activity, and VN and canonical signaling pathway expression profiles [144]. Probiotic, prebiotic, or antimicrobial administration and evaluation of food-related behaviours and metabolic outputs in healthy pregnant women are promising efforts to lower the burden of transgenerational disorders which influence the offspring’s brain development. Mechanistic studies on the GBA’s role in immune activation and CNS development have not been elucidated; however, they might provide promising targets for neurodevelopmental diseases [145]. Therefore, early fetal life is critical in terms of microbial colonization because it affects fetal physical health and is assumed to affect adult’s mental health in the long term. Furthermore, this review derived and inspired new insights into potential molecular pathways, including the molecular mechanisms of VN-mediated parasympathetic manipulation, the quantitative release and turnover of neurotransmitters in brain regions involved in neurodevelopment, and the molecular profiles in specific neural circuits responding to gut microbiota ecological dynamics, which are worthy of further investigation. Such investigations will reveal the therapeutic potential of the gut microbiota and their metabolites in preventing and treating neurological and behavioural disorders in infants and adults. Thus, combining prebiotic, vitamin, or antimicrobial administration with analysis of food-related behaviours and metabolic outputs could help reduce the therapeutic burden in healthy and unhealthy human guts.

## 8. Conclusions

As evidenced by extensive research, the GBA modulates neural signaling during the prenatal and postnatal periods. Alternatively, it is enticing to speculate whether exposure to the maternal gut microbiota and their metabolites could influence brain development during embryogenesis. Therefore, further research on the effects of maternal gut microbiota and their metabolites on neonatal brain developmental programming via the GBA is warranted. Meanwhile, in this review, we highlighted the role of maternal microbiota in the foetus prenatally and postnatally and its role in epigenetic regulation and immune responses during brain development. Following this, we discussed the critical roles of gut metabolites in intergenerational communication via the GBA. Furthermore, we briefly described the molecular characteristics of the GBA in brain development, resulting in a better understanding of neurodevelopmental disorders and therapeutic clues.

## Figures and Tables

**Table 1:** Roles of gut microbial metabolites on brain development.

Metabolites	Function	Ref.
Short-chain fatty acids (e.g., butyric acid, propionic acid, acetic acid, valeric acid, isobutyric acid, isovaleric acid, and isocaproic acid)	Modulate BBB permeability; regulate microglia activation and neuroinflammation; regulate the activity of histone deacetylase	[18] [19] [20] [21]
Amino acid metabolites (e.g., GABA, serotonin, dopamine, “TRYP6”, norepinephrine, P-cresol)	Maintain normal neurotransmission and neurodevelopment; regulate the availability of vitamin B3 and NADP+ in the brain; regulate neurotoxicity and neurodegeneration; regulate myelination and differentiation to oligodendrocytes; increase oxidative stress	[22] [23] [24] [25] [26]
Trimethylamine N oxide	Disturb mitochondria function; increase synaptic damage; promote neuroinflammation	[27] [28] [29] [30]
Polyphenolic Metabolites	Modulate neuronal receptors;antioxidation; anti-inflammation	[31] [32]
Bacterial Amyloid Proteins	Induce α-syn-aggregates in the brain; enhance neuroinflammation	[33] [34]
Cholesterol Steroids hormones	Decarboxylation; dihydroxylation Deconjugations; oxidation reductions	[35] [36]

BBB: blood-brain barrier; GABA: gamma-aminobutyric acid; NADP^+^: nicotinamide adenine dinucleotide phosphate; “trypsin-6”: six tryptophan metabolism pathways generating neuroactive metabolites, including kynurenine, quinolinate, indole, indole acetic acid, and indole propionic acid.

**Table 2:** Gut microbiota-derived neurotransmitters and their potential functions in brain development.

Gut Microbiota	Neurotransmitters	Functions	Ref
*Staphylococcus Bacillus cereus, Proteus vulgaris, Serratia marcescens,* *Escherichia coli*	Dopamine	Affect immune cells, cytokines productions by activated T cells; regulate microglial cell migration	[60,61,62,63,65]
*Escherichia coli, Bacillus subtilis, Bacillus mycoides, Proteus vulgaris,* *Serratia marcescens*	Norepinephrine	Neuroprotective effects by suppressing inflammatory genes; modulate excitatory and interneuronal responses	[66,67,68]
*Lactobacillus Bifdobacterium,* *Streptococcus*	GABA	Modulate the inhibitory balance; cytokine downregulations by proinflammatory immune cells	[69,70,71,72,73,74]
*Candida, Streptococcus, Escherichia, Enterococcus, Pseudomonas*	Serotonin	Suppress MHC class II expression; reduce proinflammatory cytokines generated by macrophages and lymphocytes; development of enteric and CNS neurons	[79,80,81,82,84,85,86]

## Data Availability

Not applicable.

## References

[1] Yamashiro K., Kurita N., Urabe T., Hattori N. (2021). Role of the Gut Microbiota in Stroke Pathogenesis and Potential Therapeutic Implications. Ann. Nutr. Metab..

[2] Chilloux J., Neves A.L., Boulangé C.L., Dumas M.E. (2016). The microbial-mammalian metabolic axis: A critical symbiotic relationship. Curr. Opin. Clin. Nutr. Metab. Care..

[3] Caricilli A.M., Saad M.J.A. (2013). The Role of Gut Microbiota on Insulin Resistance. Nutrients.

[4] Liu B.N., Liu X.T., Liang Z.H., Wang J.H. (2021). Gut microbiota in obesity. World J. Gastroenterol..

[5] Horta-Baas G., Sandoval-Cabrera A., Romero-Figueroa M.D.S. (2021). Modification of Gut Microbiota in Inflammatory Arthritis: Highlights and Future Challenges. Curr. Rheumatol. Rep..

[6] Gurung M., Li Z., You H., Rodrigues R., Jump D.B., Morgun A., Shulzhenko N. (2020). Role of gut microbiota in type 2 diabetes pathophysiology. EBioMedicine.

[7] Li J., Zhao F., Wang Y., Chen J., Tao J., Tian G., Wu S., Liu W., Cui Q., Geng B. (2017). Gut microbiota dysbiosis contributes to the development of hypertension. Microbiome.

[8] Fan Y., Pedersen O. (2021). Gut microbiota in human metabolic health and disease. Nat. Rev. Microbiol..

[9] Pluta R., Januszewski S., Czuczwar S.J. (2021). The Role of Gut Microbiota in an Ischemic Stroke. Int. J. Mol. Sci..

[10] Zhu F., Tu H., Chen T. (2022). The Microbiota–Gut–Brain Axis in Depression: The Potential Pathophysiological Mechanisms and Microbiota Combined Antidepression Effect. Nutrients.

[11] Naureen Z., Farooq S., Zahoor T., Gilani S.A. (2022). Effect of Probiotics on Gut Microbiota and Brain Interactions in the Context of Neurodegenerative and Neurodevelopmental Disorders, in Microbiome-Gut-Brain Axis.

[12] Stopińska K., Radziwoń-Zaleska M., Domitrz I. (2021). The Microbiota-Gut-Brain Axis as a Key to Neuropsychiatric Disorders: A Mini Review. J. Clin. Med..

[13] Shimada K., Nohara M., Yasuoka A., Kamei A., Shinozaki F., Kondo K., Inoue R., Kondo T., Abe K. (2022). Mouse Model of Weak Depression Exhibiting Suppressed cAMP Signaling in the Amygdala, Lower Lipid Catabolism in Liver, and Correlated Gut Microbiota. Front. Behav. Neurosci..

[14] Qin S., Liu Y., Wang S., Ma J., Yang H. (2021). Distribution characteristics of intestinal microbiota during pregnancy and postpartum in healthy women. J. Matern. Neonatal Med..

[15] Enav H., Bäckhed F., Ley R.E. (2022). The developing infant gut microbiome: A strain-level view. Cell Host Microbe.

[16] Kwon M.S., Lee H.K. (2022). Host and Microbiome Interplay Shapes the Vaginal Microenvironment. Front. Immunol..

[17] Socha-Banasiak A., Pawłowska M., Czkwianianc E., Pierzynowska K. (2021). From Intrauterine to Extrauterine Life—The Role of Endogenous and Exogenous Factors in the Regulation of the Intestinal Microbiota Community and Gut Maturation in Early Life. Front. Nutr..

[18] Sampson T.R., Mazmanian S.K. (2015). Control of Brain Development, Function, and Behavior by the Microbiome. Cell Host Microbe.

[19] Braniste V., Al-Asmakh M., Kowal C., Anuar F., Abbaspour A., Tóth M., Korecka A., Bakocevic N., Ng L.G., Kundu P. (2014). The gut microbiota influences blood-brain barrier permeability in mice. Sci. Transl. Med..

[20] Wenzel T.J., Gates E.J., Ranger A.L., Klegeris A. (2020). Short-chain fatty acids (SCFAs) alone or in combination regulate select immune functions of microglia-like cells. Mol. Cell. Neurosci..

[21] Stilling R.M., van de Wouw M., Clarke G., Stanton C., Dinan T.G., Cryan J.F. (2016). The neuropharmacology of butyrate: The bread and butter of the microbiota-gut-brain axis?. Neurochem. Int..

[22] Gill S.R., Pop M., DeBoy R.T., Eckburg P.B., Turnbaugh P.J., Samuel B.S., Gordon J.I., Relman D.A., Fraser-Liggett C.M., Nelson K.E. (2006). Metagenomic Analysis of the Human Distal Gut Microbiome. Science.

[23] Parthasarathy A., Cross P.J., Dobson R.C.J., Adams L.E., Savka M.A., Hudson A.O. (2018). A Three-Ring Circus: Metabolism of the Three Proteogenic Aromatic Amino Acids and Their Role in the Health of Plants and Animals. Front. Mol. Biosci..

[24] Kang D.-W., Ilhan Z.E., Isern N.G., Hoyt D.W., Howsmon D.P., Shaffer M., Lozupone C.A., Hahn J., Adams J.B., Krajmalnik-Brown R. (2018). Differences in fecal microbial metabolites and microbiota of children with autism spectrum disorders. Anaerobe.

[25] Song S., Liu J., Zhang F., Hong J.-S. (2020). Norepinephrine depleting toxin DSP-4 and LPS alter gut microbiota and induce neurotoxicity in α-synuclein mutant mice. Sci. Rep..

[26] Shandilya S., Kumar S., Jha N.K., Kesari K.K., Ruokolainen J. (2021). Interplay of gut microbiota and oxidative stress: Perspective on neurodegeneration and neuroprotection. J. Adv. Res..

[27] Li D., Ke Y., Zhan R., Liu C., Zhao M., Zeng A., Shi X., Ji L., Cheng S., Pan B. (2018). Trimethylamine-*N* -oxide promotes brain aging and cognitive impairment in mice. Aging Cell.

[28] Govindarajulu M., Pinky P.D., Steinke I., Bloemer J., Ramesh S., Kariharan T., Rella R.T., Bhattacharya S., Dhanasekaran M., Suppiramaniam V. (2020). Gut Metabolite TMAO Induces Synaptic Plasticity Deficits by Promoting Endoplasmic Reticulum Stress. Front. Mol. Neurosci..

[29] Brunt V.E., LaRocca T.J., Bazzoni A.E., Sapinsley Z.J., Miyamoto-Ditmon J., Gioscia-Ryan R.A., Neilson A.P., Link C.D., Seals D.R. (2020). The gut microbiome–derived metabolite trimethylamine N-oxide modulates neuroinflammation and cognitive function with aging. GeroScience.

[30] Filosa S., Di Meo F., Crispi S. (2018). Polyphenols-gut microbiota interplay and brain neuromodulation. Neural Regen. Res..

[31] Feng X., Li Y., Oppong M.B., Qiu F. (2018). Insights into the intestinal bacterial metabolism of flavonoids and the bioactivities of their microbe-derived ring cleavage metabolites. Drug Metab. Rev..

[32] Gasperotti M., Passamonti S., Tramer F., Masuero D., Guella G., Mattivi F., Vrhovsek U. (2015). Fate of microbial metabolites of dietary polyphenols in rats: Is the brain their target destination?. ACS Chem. Neurosci..

[33] Friedland R.P., Chapman M.R. (2017). The role of microbial amyloid in neurodegeneration. PLOS Pathog..

[34] Sampson T.R., Challis C., Jain N., Moiseyenko A., Ladinsky M.S., Shastri G.G., Thron T., Needham B.D., Horvath I., Debelius J.W. (2020). A gut bacterial amyloid promotes α-synuclein aggregation and motor impairment in mice. eLife.

[35] Needham B.D., Kaddurah-Daouk R., Mazmanian S.K. (2020). Gut microbial molecules in behavioural and neurodegenerative conditions. Nat. Rev. Neurosci..

[36] DiviDiviccaro S., FitzGerald J.A., Cioffi L., Falvo E., Crispie F., Cotter P.D., O’Mahony S.M., Giatti S., Caruso D., Melcangi R.C. (2022). Gut Steroids and Microbiota: Effect of Gonadectomy and Sex. Biomolecules.

[37] Kanai T., Teratani T. (2022). Role of the Vagus Nerve in the Gut-Brain Axis: Development and Maintenance of Gut Regulatory T Cells via the Liver-Brain-Gut Vago-Vagal Reflex. Brain Nerve.

[38] Washburn B.S., Jiang J.C., Cummings S.L., Dixon K., Gietzen D.W. (1994). Anorectic responses to dietary amino acid imbalance: Effects of vagotomy and tropisetron. Am. J. Physiol. Integr. Comp. Physiol..

[39] Rutsch A., Kantsjö J.B., Ronchi F. (2020). The Gut-Brain Axis: How Microbiota and Host Inflammasome Influence Brain Physiology and Pathology. Front. Immunol..

[40] AuAustelle C.W., Ba G.H.O., Thompson S., Gruber E., Kahn A., Manett A.J., Short B., Badran B.W., Bs S.T., Bs E.G. (2021). A Comprehensive Review of Vagus Nerve Stimulation for Depression. Neuromodulation: Technol. Neural Interface.

[41] HummHummel G.L., Austin K., Cunningham-Hollinger H.C. (2022). Comparing the maternal-fetal microbiome of humans and cattle: A translational assessment of the reproductive, placental, and fetal gut microbiomes. Biol. Reprod..

[42] Celentano C., Matarrelli B., Pavone G., Vitacolonna E., Mattei P.A., Berghella V., Liberati M. (2018). The influence of different inositol stereoisomers supplementation in pregnancy on maternal gestational diabetes mellitus and fetal outcomes in high-risk patients: A randomized controlled trial. J. Matern. Neonatal Med..

[43] Manta-Vogli P.D., Schulpis K.H., Dotsikas Y., Yannis L. (2018). The significant role of amino acids during pregnancy: Nutritional support. J. Matern. Neonatal Med..

[44] Li P., Tang T., Chang X., Fan X., Chen X., Wang R., Fan C., Qi K. (2018). Abnormality in Maternal Dietary Calcium Intake During Pregnancy and Lactation Promotes Body Weight Gain by Affecting the Gut Microbiota in Mouse Offspring. Mol. Nutr. Food Res..

[45] JanJandhyala S.M., Talukdar R., Subramanyam C., Vuyyuru H., Sasikala M., Nageshwar Reddy D. (2015). Role of the normal gut microbiota. World J. Gastroenterol..

[46] Saraf V.S., Sheikh S.A., Ahmad A., Gillevet P.M., Bokhari H., Javed S. (2021). Vaginal microbiome: Normalcy vs dysbiosis. Arch. Microbiol..

[47] Shi H., Ge X., Ma X., Zheng M., Cui X., Pan W., Zheng P., Yang X., Hu M., Hu T. (2021). A fiber-deprived diet causes cognitive impairment and hippocampal microglia-mediated synaptic loss through the gut microbiota and metabolites. Microbiome.

[48] Butel M.-J., Waligora-Dupriet A.-J., Wydau-Dematteis S. (2018). The developing gut microbiota and its consequences for health. J. Dev. Orig. Health Dis..

[49] NeNeuman H., Koren O. (2017). The Pregnancy Microbiome. Nestlé Nutr. Inst. Workshop Ser..

[50] Greenhalgh K., Meyer K.M., Aagaard K.M., Wilmes P. (2016). The human gut microbiome in health: Establishment and resilience of microbiota over a lifetime. Environ. Microbiol..

[51] Cirulli F., Musillo C., Berry A. (2020). Maternal Obesity as a Risk Factor for Brain Development and Mental Health in the Offspring. Neuroscience.

[52] Cerdó T., Diéguez E., Campoy C. (2019). Early nutrition and gut microbiome: Interrelationship between bacterial metabolism, immune system, brain structure, and neurodevelopment. Am. J. Physiol. Metab..

[53] Claesson M.J., Wang Q., O’Sullivan O., Greene-Diniz R., Cole J.R., Ross R., O’Toole P.W. (2010). Comparison of two next-generation sequencing technologies for resolving highly complex microbiota composition using tandem variable 16S rRNA gene regions. Nucleic Acids Res..

[54] Rodríguez J.M., Murphy K., Stanton C., Ross R.P., Kober O.I., Juge N., Avershina E., Rudi K., Narbad A., Jenmalm M.C. (2015). The composition of the gut microbiota throughout life, with an emphasis on early life. Microbes Ecol. Health Dis..

[55] Dougherty M.W., Kudin O., Mühlbauer M., Neu J., Gharaibeh R.Z., Jobin C. (2020). Gut microbiota maturation during early human life induces enterocyte proliferation via microbial metabolites. BMC Microbiol..

[56] Chen X., Yan Z., Liu L., Zhang R., Zhang X., Peng C., Geng Y., Zhou F., Han Y., Hou X. (2022). Characteristics of gut microbiota of term small gestational age infants within 1 week and their relationship with neurodevelopment at 6 months. Front. Microbiol..

[57] ThThion M.S., Low D., Silvin A., Chen J., Grisel P., Schulte-Schrepping J., Blecher R., Ulas T., Squarzoni P., Hoeffel G. (2017). Microbiome Influences Prenatal and Adult Microglia in a Sex-Specific Manner. Cell.

[58] Vuong H.E., Pronovost G.N., Williams D.W., Coley E.J.L., Siegler E.L., Qiu A., Kazantsev M., Wilson C.J., Rendon T., Hsiao E.Y. (2020). The maternal microbiome modulates fetal neurodevelopment in mice. Nature.

[59] Eisenhofer G., Kopin I.J., Goldstein D.S. (2004). Catecholamine Metabolism: A Contemporary View with Implications for Physiology and Medicine. Pharmacol. Rev..

[60] Luqman A., Nega M., Nguyen M.T., Ebner P., Götz F. (2018). SadA-Expressing Staphylococci in the Human Gut Show Increased Cell Adherence and Internalization. Cell Rep..

[61] Roshchina V.V. (2016). New Trends and Perspectives in the Evolution of Neurotransmitters in Microbial, Plant, and Animal Cells. Adv. Exp. Med. Biol..

[62] SarSarkar C., Basu B., Chakroborty D., Dasgupta P.S., Basu S. (2010). The immunoregulatory role of dopamine: An update. Brain, Behav. Immun..

[63] Färber K., Pannasch U., Kettenmann H. (2005). Dopamine and noradrenaline control distinct functions in rodent microglial cells. Mol. Cell. Neurosci..

[64] Chang J.Y., Liu L.-Z. (2000). Catecholamines inhibit microglial nitric oxide production. Brain Res. Bull..

[65] Borodovitsyna O., Flamini M., Chandler D. (2017). Noradrenergic Modulation of Cognition in Health and Disease. Neural Plast..

[66] Tsavkelova E.A., Botvinko I.V., Kudrin V.S., Oleskin A.V. (2000). Detection of neurotransmitter amines in microorganisms with the use of high-performance liquid chromatography. Dokl. Biochem..

[67] Zafra F., Lindholm D., Castrén E., Hartikka J., Thoenen H. (1992). Regulation of brain-derived neurotrophic factor and nerve growth factor mRNA in primary cultures of hippocampal neurons and astrocytes. J. Neurosci..

[68] O’Donnell J., Zeppenfeld D., McConnell E., Pena S., Nedergaard M. (2012). Norepinephrine: A Neuromodulator That Boosts the Function of Multiple Cell Types to Optimize CNS Performance. Neurochem. Res..

[69] Barrett E., Ross R.P., O’Toole P.W., Fitzgerald G.F., Stanton C. (2012). γ-Aminobutyric acid production by culturable bacteria from the human intestine. J. Appl. Microbiol..

[70] Takanaga H., Ohtsuki S., Hosoya K.I., Terasaki T. (2001). GAT2/BGT-1 as a system responsible for the transport of gamma-aminobutyric acid at the mouse blood-brain barrier. J. Cereb. Blood Flow Metab..

[71] Al-Sarraf H. (2002). Transport of 14C-γ-aminobutyric acid into brain, cerebrospinal fluid and choroid plexus in neonatal and adult rats. Dev. Brain Res..

[72] Shyamaladevi N., Jayakumar A.R., Sujatha R., Paul V., Subramanian E.H. (2002). Evidence that nitric oxide production increases gamma-amino butyric acid permeability of blood-brain barrier. Brain Res. Bull..

[73] Bjurstöm H., Wang J., Ericsson I., Bengtsson M., Liu Y., Mendu S.K., Issazadeh-Navikas S., Birnir B. (2008). GABA, a natural immunomodulator of T lymphocytes. J. Neuroimmunol..

[74] Wu C., Sun D. (2015). GABA receptors in brain development, function, and injury. Metab. Brain Dis..

[75] Krantis A. (2000). GABA in the Mammalian Enteric Nervous System. News Physiol. Sci. Int. J. Physiol. Prod. jointly by Int. Union Physiol. Sci. Am. Physiol. Soc..

[76] Bhat R., Axtell R., Mitra A., Miranda M., Lock C., Tsien R.W., Steinman L. (2010). Inhibitory role for GABA in autoimmune inflammation. Proc. Natl. Acad. Sci. USA.

[77] Wikoff W.R., Anfora A.T., Liu J., Schultz P.G., Lesley S.A., Peters E.C., Siuzdak G. (2009). Metabolomics analysis reveals large effects of gut microflora on mammalian blood metabolites. Proc. Natl. Acad. Sci. USA.

[78] Heijtz R.D., Wang S., Anuar F., Qian Y., Björkholm B., Samuelsson A., Hibberd M.L., Forssberg H., Pettersson S. (2011). Normal gut microbiota modulates brain development and behavior. Proc. Natl. Acad. Sci. USA.

[79] Evrensel A., Ceylan M.E. (2015). The Gut-Brain Axis: The Missing Link in Depression. Clin. Psychopharmacol. Neurosci.

[80] Abbott N.J. (2000). Inflammatory Mediators and Modulation of Blood–Brain Barrier Permeability. Cell. Mol. Neurobiol..

[81] Sternberg E.M., Wedner H.J., Leung M.K., Parker C.W. (1987). Effect of serotonin (5-HT) and other monoamines on murine macrophages: Modulation of interferon-gamma induced phagocytosis. J. Immunol..

[82] Kubera M., Maes M., Kenis G., Kim Y.-K., Lasoń W. (2005). Effects of serotonin and serotonergic agonists and antagonists on the production of tumor necrosis factor α and interleukin-6. Psychiatry Res..

[83] Bellono N.W., Bayrer J.R., Leitch D.B., Castro J., Zhang C., O’Donnell T.A., Brierley S.M., Ingraham H.A., Julius D. (2017). Enterochromaffin Cells Are Gut Chemosensors that Couple to Sensory Neural Pathways. Cell.

[84] Liu M.-T., Kuan Y.-H., Wang J., Hen R., Gershon M.D. (2009). 5-HT4 receptor-mediated neuroprotection and neurogenesis in the enteric nervous system of adult mice. J. Neurosci..

[85] McVey Neufeld K.A., Perez-Burgos A., Mao Y.K., Bienenstock J., Kunze W.A. (2015). The gut microbiome restores intrinsic and extrinsic nerve function in germ-free mice accompanied by changes in calbindin. Neurogastroenterol. Motil..

[86] Jiang S.-H., Li J., Dong F.-Y., Yang J.-Y., Liu D.-J., Yang X.-M., Wang Y.-H., Yang M.-W., Fu X.-L., Zhang X.-X. (2017). Increased Serotonin Signaling Contributes to the Warburg Effect in Pancreatic Tumor Cells Under Metabolic Stress and Promotes Growth of Pancreatic Tumors in Mice. Gastroenterology.

[87] Sánchez-Romero M.A., Cota I., Casadesús J. (2015). DNA methylation in bacteria: From the methyl group to the methylome. Curr. Opin. Microbiol..

[88] Wiseman A.K., Tiedemann R.L., Fan H., Shen H., Madaj Z., McCabe M.T., Pappalardi M.B., Jones P.A. (2022). Chromosome-specific retention of cancer-associated DNA hypermethylation following pharmacological inhibition of DNMT1. Commun. Biol..

[89] Omar N.N., Mosbah R.A., Sarawi W.S., Rashed M.M., Badr A.M. (2022). Rifaximin Protects against Malathion-Induced Rat Testicular Toxicity: A Possible Clue on Modulating Gut Microbiome and Inhibition of Oxidative Stress by Mitophagy. Molecules.

[90] Upadhyay T.K., Goel H., Goyal K., Pandey A.K., Benjamin M., Khan F., Pandey P., Mittan S., Iqbal D., Alsaweed M. (2023). Elucidations of Molecular Mechanism and Mechanistic Effects of Environmental Toxicants in Neurological Disorders. CNS Neurol. Disord.—Drug Targets.

[91] Li D., Li Y., Yang S., Lu J., Jin X., Wu M. (2022). Diet-gut microbiota-epigenetics in metabolic diseases: From mechanisms to therapeutics. Biomed. Pharmacother..

[92] Paquette A.G., Houseman E.A., Green B.B., Lesseur C., Armstrong D.A., Lester B., Marsit C.J. (2016). Regions of variable DNA methylation in human placenta associated with newborn neurobehavior. Epigenetics.

[93] Tung P.W., Burt A., Karagas M., Jackson B.P., Punshon T., Lester B., Marsit C.J. (2021). Association between placental toxic metal exposure and NICU Network Neurobehavioral Scales (NNNS) profiles in the Rhode Island Child Health Study (RICHS). Environ. Res..

[94] Oakley R.H., Whirledge S.D., Petrillo M.G., Riddick N.V., Xu X., Moy S.S., Cidlowski J.A. (2021). Combinatorial actions of glucocorticoid and mineralocorticoid stress hormone receptors are required for preventing neurodegeneration of the mouse hippocampus. Neurobiol. Stress.

[95] Stroud L.R., Papandonatos G.D., Salisbury A.L., Phipps M.G., Huestis M.A., Niaura R., Padbury J.F., Marsit C.J., Lester B.M. (2016). Epigenetic Regulation of Placental NR3C1, Mechanism Underlying Prenatal Programming of Infant Neurobehavior by Maternal Smoking?. Child Dev..

[96] Cukrowska B., Bierła J.B., Zakrzewska M., Klukowski M., Maciorkowska E. (2020). The Relationship between the Infant Gut Microbiota and Allergy. The Role of Bifidobacterium breve and Prebiotic Oligosaccharides in the Activation of Anti-Allergic Mechanisms in Early Life. Nutrients.

[97] Barcik W., Boutin R.C.T., Sokolowska M., Finlay B.B. (2020). The Role of Lung and Gut Microbiota in the Pathology of Asthma. Immunity.

[98] Zhang B., Ren D., Zhao A., Cheng Y., Liu Y., Zhao Y., Yang X. (2022). *Eurotium cristatum* reduces obesity by alleviating gut microbiota dysbiosis and modulating lipid and energy metabolism. J. Sci. Food Agric..

[99] Nishida A., Inoue R., Inatomi O., Bamba S., Naito Y., Andoh A. (2017). Gut microbiota in the pathogenesis of inflammatory bowel disease. Clin. J. Gastroenterol..

[100] Caio G., Lungaro L., Segata N., Guarino M., Zoli G., Volta U., de Giorgio R. (2020). Effect of Gluten-Free Diet on Gut Microbiota Composition in Patients with Celiac Disease and Non-Celiac Gluten/Wheat Sensitivity. Nutrients.

[101] Dabke K., Hendrick G., Devkota S. (2019). The gut microbiome and metabolic syndrome. J. Clin. Investig..

[102] Tang W.W., Kitai T., Hazen S.L. (2017). Gut Microbiota in Cardiovascular Health and Disease. Circ. Res..

[103] Fendrich S.J., Koralnik L.R., Bonner M., Goetz D., Joe P., Lee J., Mueller B., Robinson-Papp J., Gonen O., Clemente J.C. (2022). Patient-reported exposures and outcomes link the gut-brain axis and inflammatory pathways to specific symptoms of severe mental illness. Psychiatry Res..

[104] Nandwana V., Nandwana N.K., Das Y., Saito M., Panda T., Das S., Almaguel F., Hosmane N.S., Das B.C. (2022). The Role of Microbiome in Brain Development and Neurodegenerative Diseases. Molecules.

[105] Sharp G.C., Lawlor D.A., Richardson S.S. (2018). It’s the mother!: How assumptions about the causal primacy of maternal effects influence research on the developmental origins of health and disease. Soc. Sci. Med..

[106] Jašarević E., Howard C.D., Misic A.M., Beiting D.P., Bale T.L. (2017). Stress during pregnancy alters temporal and spatial dynamics of the maternal and offspring microbiome in a sex-specific manner. Sci. Rep..

[107] Duranti S., Ruiz L., Lugli G.A., Tames H., Milani C., Mancabelli L., Mancino W., Longhi G., Carnevali L., Sgoifo A. (2020). Bifidobacterium adolescentis as a key member of the human gut microbiota in the production of GABA. Sci. Rep..

[108] Miyata S., Kumagaya R., Kakizaki T., Fujihara K., Wakamatsu K., Yanagawa Y. (2019). Loss of Glutamate Decarboxylase 67 in Somatostatin-Expressing Neurons Leads to Anxiety-Like Behavior and Alteration in the Akt/GSK3β Signaling Pathway. Front. Behav. Neurosci..

[109] Zhao F., Wang K., Wen Y., Chen X., Liu H., Qi F., Fu Y., Zhu J., Guan S., Liu Z. (2022). Contribution of hippocampal BDNF/CREB signaling pathway and gut microbiota to emotional behavior impairment induced by chronic unpredictable mild stress during pregnancy in rats offspring. PeerJ.

[110] Hoffman K.W., Lee J.J., Corcoran C.M., Kimhy D., Kranz T.M., Malaspina D. (2020). Considering the Microbiome in Stress-Related and Neurodevelopmental Trajectories to Schizophrenia. Front. Psychiatry.

[111] Sanguinetti E., Guzzardi M.A., Tripodi M., Panetta D., Selma-Royo M., Zega A., Telleschi M., Collado M.C., Iozzo P. (2019). Microbiota signatures relating to reduced memory and exploratory behaviour in the offspring of overweight mothers in a murine model. Sci. Rep..

[112] Lohuis M.A.M., Werkman C.C.N., Harmsen H.J.M., Tietge U.J.F., Verkade H.J. (2018). Absence of Intestinal Microbiota during Gestation and Lactation Does Not Alter the Metabolic Response to a Western-type Diet in Adulthood. Mol. Nutr. Food Res..

[113] Kawano Y., Edwards M., Huang Y., Bilate A.M., Araujo L.P., Tanoue T., Atarashi K., Ladinsky M.S., Reiner S.L., Wang H.H. (2022). Microbiota imbalance induced by dietary sugar disrupts immune-mediated protection from metabolic syndrome. Cell.

[114] Barouei J., Bendiks Z., Martinic A., Mishchuk D., Heeney D., Hsieh Y.-H., Kieffer D., Zaragoza J., Martin R., Slupsky C. (2017). Microbiota, metabolome, and immune alterations in obese mice fed a high-fat diet containing type 2 resistant starch. Mol. Nutr. Food Res..

[115] Ivanov I.I., Atarashi K., Manel N., Brodie E.L., Shima T., Karaoz U., Wei D., Goldfarb K.C., Santee C.A., Lynch S.V. (2009). Induction of intestinal Th17 cells by segmented filamentous bacteria. Cell.

[116] Kim S., Kim H., Yim Y.S., Ha S., Atarashi K., Tan T.G., Longman R.S., Honda K., Littman D.R., Choi G.B. (2017). Maternal gut bacteria promote neurodevelopmental abnormalities in mouse offspring. Nature.

[117] del Pozo-Acebo L., Hazas M.L.D.L., Margollés A., Dávalos A., García-Ruiz A. (2021). Eating microRNAs: Pharmacological opportunities for cross-kingdom regulation and implications in host gene and gut microbiota modulation. J. Cereb. Blood Flow Metab..

[118] Jašarević E., Bale T.L. (2019). Prenatal and postnatal contributions of the maternal microbiome on offspring programming. Front. Neuroendocr..

[119] Coley E.J., Hsiao E.Y. (2021). Malnutrition and the microbiome as modifiers of early neurodevelopment. Trends Neurosci..

[120] Sun X., Fukami T., Li T., Desai M., Ross M.G. (2016). Preferential development of neuropeptide Y/GABA circuit in hypothalamic arcuate nucleus in postnatal rats. Brain Res..

[121] Arruda-Carvalho M., Wu W.-C., Cummings K.A., Clem R.L. (2017). Optogenetic Examination of Prefrontal-Amygdala Synaptic Development. J. Neurosci..

[122] Biagi E., Quercia S., Aceti A., Beghetti I., Rampelli S., Turroni S., Faldella G., Candela M., Brigidi P., Corvaglia L. (2017). The Bacterial Ecosystem of Mother’s Milk and Infant’s Mouth and Gut. Front. Microbiol..

[123] Socała K., Doboszewska U., Szopa A., Serefko A., Włodarczyk M., Zielińska A., Poleszak E., Fichna J., Wlaź P. (2021). The role of microbiota-gut-brain axis in neuropsychiatric and neurological disorders. Pharmacol. Res..

[124] Marques T.M., Wall R., Ross R.P., Fitzgerald G.F., Ryan C.A., Stanton C. (2010). Programming infant gut microbiota: Influence of dietary and environmental factors. Curr. Opin. Biotechnol..

[125] Zubair M., Fatima F., Husain F.M. (2022). Behavioral Abnormalities of Gut Microbiota and Progression of Dementia. Current Thoughts on Dementia.

[126] Glenny E.M., Fouladi F., Thomas S.A., Bulik-Sullivan E.C., Tang Q., Djukic Z., Trillo-Ordonez Y.S., Fodor A.A., Tarantino L.M., Bulik C.M. (2021). Gut microbial communities from patients with anorexia nervosa do not influence body weight in recipient germ-free mice. Gut Microbes.

[127] Liu W.-H., Chuang H.-L., Huang Y.-T., Wu C.-C., Chou G.-T., Wang S., Tsai Y.-C. (2015). Alteration of behavior and monoamine levels attributable to Lactobacillus plantarum PS128 in germ-free mice. Behav. Brain Res..

[128] Faucher P., Dries A., Mousset P., Leboyer M., Dore J., Beracochea D. (2022). Synergistic effects of *Lacticaseibacillus rhamnosus* GG, glutamine, and curcumin on chronic unpredictable mild stress-induced depression in a mouse model. Benef. Microbes.

[129] Dinan T.G., Cryan J.F. (2016). Gut instincts: Microbiota as a key regulator of brain development, ageing and neurodegeneration. J. Physiol..

[130] Srikantha P., Mohajeri M.H. (2019). The Possible Role of the Microbiota-Gut-Brain-Axis in Autism Spectrum Disorder. Int. J. Mol. Sci..

[131] Yue Q., Cai M., Xiao B., Zhan Q., Zeng C. (2022). The Microbiota-Gut-Brain Axis and Epilepsy. Cell. Mol. Neurobiol..

[132] Wu J., Zhang Y., Yang H., Rao Y., Miao J., Lu X. (2016). Intestinal Microbiota as an Alternative Therapeutic Target for Epilepsy. Can. J. Infect. Dis. Med Microbiol..

[133] Kim E., Paik D., Ramirez R.N., Biggs D.G., Park Y., Kwon H.-K., Choi G.B., Huh J.R. (2021). Maternal gut bacteria drive intestinal inflammation in offspring with neurodevelopmental disorders by altering the chromatin landscape of CD4+ T cells. Immunity.

[134] Sililas P., Huang L., Thonusin C., Luewan S., Chattipakorn N., Chattipakorn S., Tongsong T. (2021). Association between Gut Microbiota and Development of Gestational Diabetes Mellitus. Microorganisms.

[135] Tang R., Xiao G., Jian Y., Yuan Q., Jiang C., Wang W. (2022). The Gut Microbiota Dysbiosis in Preeclampsia Contributed to Trophoblast Cell Proliferation, Invasion, and Migration via lncRNA BC030099/NF-κB Pathway. Mediat. Inflamm..

[136] Gilley S.P., Ruebel M.L., Sims C., Zhong Y., Turner D., Lan R.S., Pack L.M., Piccolo B.D., Chintapalli S.V., Abraham A. (2022). Associations between maternal obesity and offspring gut microbiome in the first year of life. Pediatr. Obes..

[137] Kar F., Hacioglu C., Kar E., Donmez D.B., Kanbak G. (2022). Probiotics ameliorates LPS induced neuroinflammation injury on Aβ 1–42, APP, γ-β secretase and BDNF levels in maternal gut microbiota and fetal neurodevelopment processes. Metab. Brain Dis..

[138] Saunders J.M., Moreno J.L., Ibi D., Sikaroodi M., Kang D.J., Muñoz-Moreno R., Dalmet S.S., García-Sastre A., Gillevet P.M., Dozmorov M.G. (2020). Gut microbiota manipulation during the prepubertal period shapes behavioral abnormalities in a mouse neurodevelopmental disorder model. Sci. Rep..

[139] Pan Y.-Q., Zheng Q.-X., Jiang X.-M., Chen X.-Q., Zhang X.-Y., Wu J.-L. (2021). Probiotic Supplements Improve Blood Glucose and Insulin Resistance/Sensitivity among Healthy and GDM Pregnant Women: A Systematic Review and Meta-Analysis of Randomized Controlled Trials. Evidence-Based Complement. Altern. Med..

[140] Lacroix M., Kina E., Hivert M.-F. (2013). Maternal/Fetal Determinants of Insulin Resistance in Women During Pregnancy and in Offspring Over Life. Curr. Diabetes Rep..

[141] Choudhury A.A., Rajeswari V.D. (2021). Gestational diabetes mellitus—A metabolic and reproductive disorder. Biomed. Pharmacother..

[142] Margolis K.G., Cryan J.F., Mayer E.A. (2021). The Microbiota-Gut-Brain Axis: From Motility to Mood. Gastroenterology.

[143] Wang S., Harvey L., Martin R., van der Beek E.M., Knol J., Cryan J.F., Renes I.B. (2018). Targeting the gut microbiota to influence brain development and function in early life. Neurosci. Biobehav. Rev..

[144] Chalazonitis A., Rao M., Sulzer D. (2022). Similarities and differences between nigral and enteric dopaminergic neurons unravel distinctive involvement in Parkinson’s disease. NPJ Park. Dis..

[145] Alves J.L.D.B., de Oliveira Y., Carvalho N.N.C., Cavalcante R.G.S., Lira M.M.P., Nascimento L.C.P.D., Magnani M., Vidal H., Braga V.D.A., de Souza E.L. (2019). Gut microbiota and probiotic intervention as a promising therapeutic for pregnant women with cardiometabolic disorders: Present and future directions. Pharmacol. Res..

